# Prevalence of SARS-CoV-2 infection in neonates born to mothers or relatives with COVID-19

**DOI:** 10.1186/s12879-022-07688-6

**Published:** 2022-09-08

**Authors:** Roghayeh Babaei, Farah Bokharaei-Salim, Khadijeh Khanaliha, Seyed Jalal Kiani, Arezoo Marjani, Saba Garshasbi, Farzaneh Dehghani-Dehej, Sara Chavoshpour

**Affiliations:** 1 Department of Medical Nanotechnology, Faculty of Advanced Sciences and Technology, Tehran Medical Sciences, Islamic Azad University, Tehran, Iran; 2 Department of Virology, School of Medicine, Iran University of Medical Sciences (IUMS), Tehran, Iran; 3 Research Center of Pediatric Infectious Diseases, Institute of Immunology and Infectious Diseases, Iran University of Medical Sciences (IUMS), Tehran, Iran; 4 Department of Virology, School of Public Health, Tehran University of Medical Sciences (TUMS), Tehran, Iran; 5 Department of Molecular Medicine, Faculty of Advanced Technologies in Medicine, Iran University of Medical Sciences, Tehran, Iran; 6 Deputy of Health, Iran University of Medical Sciences (IUMS), Tehran, Iran

**Keywords:** COVID-19, SARS-CoV-2 infection, Coronavirus, Neonates, Pregnant women, Prevalence

## Abstract

**Background:**

In December 2019, in Wuhan, China, coronavirus disease 2019 (COVID-19) was emerged due to severe acute respiratory syndrome coronavirus-2 (SARS-CoV-2). It seems that children and neonates, similar to adult and elderly individuals, are at risk of SARS-CoV-2 infection. However, adequate data are not available about neonates infected with SARS-CoV-2.

**Methods:**

This study evaluated the presence of SARS-CoV-2 infection in neonates born to mothers or relatives with COVID-19. This cross-sectional study was performed on 25,044 consecutive Iranian participants in Tehran, Iran, from January 2020 to August 2020. Viral ribonucleic acid (RNA) was extracted from 500 µl of the oropharyngeal and nasopharyngeal specimens of the participants. The genomic RNA of SARS-CoV-2 was detected by real-time polymerase chain reaction (PCR) assay.

**Results:**

Out of all participants, 98 (0.40%) cases were neonates born to mothers or relatives with SARS-CoV-2 infection. Therefore, the current study was performed on these neonates. Out of 98 studied neonates, 6 (6.1%) cases had positive PCR results for SARS-CoV-2 infection. Moreover, among 98 studied neonates’ mothers, 25 (25.5%) cases had positive PCR results for SARS-CoV-2 infection.

**Conclusion:**

The findings of this study demonstrated that the rate of COVID-19 in neonates born to mothers or relatives with SARS-CoV-2 infection in the Iranian population is about 6.1%.

## Background

To date, some species of coronavirus, including NL63, 229E, HKU1, and OC43, have been identified as causing disease in humans with cold symptoms [1]. Other zoonotic species belonging to the *Coronaviridae* family, such as Middle East respiratory syndrome coronavirus (MERS-CoV) and severe acute respiratory syndrome coronavirus (SARS-CoV), which have been circulating between humans and animals, have caused severe respiratory diseases [2]. In December 2019, after identifying individuals with severe pneumonia in Wuhan, China, a new species of the *Coronaviridae* family was discovered, which was afterward named severe acute respiratory syndrome coronavirus-2 (SARS-CoV-2), causing coronavirus disease 2019 (COVID-19) [3–5]. Different age groups are exposed to COVID-19; however, it seems that the elderly and individuals with underlying medical conditions experience a severe and life-threatening form of COVID-19. However, children have been shown to have fewer clinical symptoms after being infected with SARS-CoV-2 [6, 7]. In other words, the findings showed that children experience less severe effects as a result of SARS-CoV-2 infection than adults [8]. On the other hand, accurate information about the effect of SARS-CoV-2 on neonates during the first month after birth is not available.

It is noteworthy that neonates are not similarly exposed to SARS-CoV-2 as older individuals. In addition to the fact that neonates, similar to other age groups, are exposed to SARS-CoV-2 through close contact, they are also more likely to be infected with the virus through vertical transmission during pregnancy or at birth [9].

Furthermore, the immune system response to infection in neonates is immature. On the other hand, defense mechanisms are not similar as in adults. Therefore, they may be very susceptible to infectious diseases. The infrequent data have demonstrated that neonates and infants are more likely to develop the severe illness than older children [10–14]. The damage caused by COVID-19 in infants and neonates is not fully understood [15]. Recent findings have shown that no intrauterine infection due to vertical transmission has been observed in pregnant women with COVID-19 [16]. Nevertheless, the vertical transmission of SARS-CoV-2 from mother to fetus is an important and controversial issue [17]. Due to severe conditions in pregnant women and high mortality rates due to SARS-CoV and MERS-CoV, SARS-CoV-2 has caused considerable concern for pregnant women and their neonates [18–21]. In addition, susceptibility to infection and consequent hypoxia has been observed in pregnant women and their neonates [22–24].

In some countries, including China, it is suggested to separate newborns from mothers infected with SARS-CoV-2 and even prevent breastfeeding [25]. However, the World Health Organization (WHO) suggests that mothers with COVID-19 start and continue breastfeeding. It is better for the mothers and their neonates to stay in a common place together. Furthermore, the WHO emphasized that it is necessary to observe hygiene precautions [26, 27]. Significantly, the process of the separation of mothers and their newborns likely leads to psychological problems in the mother-neonate relationship [28].

To date, the required information on the status of neonates infected with SARS-CoV-2 is not available. Regarding the transmission of SARS-CoV-2 from mother to neonate and the lack of sufficient information about COVID-19 in neonates, the guidelines for the management of pregnant mothers and newborns at risk of SARS-CoV-2 have significantly changed [25, 29]. The current survey assessed the presence of SARS-CoV-2 infection in neonates born to mothers or relatives with COVID-19.

## Methods and patients

### Study populations

**Fig. 1:**
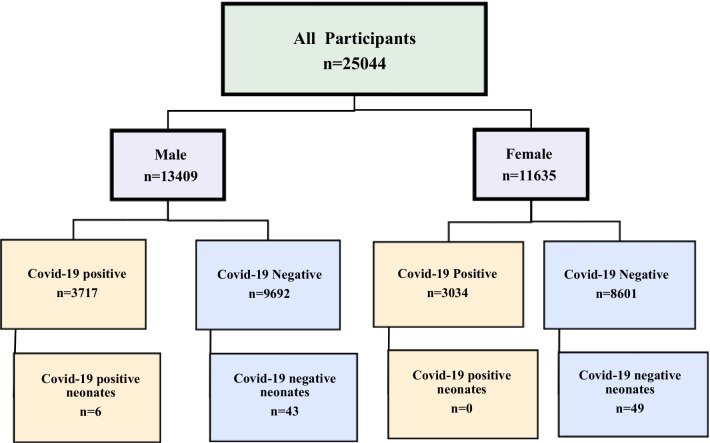
All results about the molecular virology assessment for SARS-CoV-2 infection in all studied participants

From January 2020 to August 2020, 25,044 consecutive Iranian were studied in this cross-sectional survey (Fig. 1). These people had referred to one of the hospitals or clinics affiliated to Iran University of Medical Sciences (IUMS), Tehran, Iran. Of those studied, 98 (0.4%) were neonates born to mothers or all those who live together in the same house and are in contact with the neonates with COVID-19, and the present study was performed on these neonates.

### Collection of the specimens and RNA extraction

To diagnose infection with SARS-CoV-2, the nasopharyngeal and oropharyngeal specimens were taken from the participants and placed in a viral transport media (VTM) and sent the samples to a molecular diagnostic laboratory of IUMS. It is noteworthy that only oropharyngeal samples were taken from the neonates. The viral RNA was extracted from 500 µl of the nasopharyngeal and oropharyngeal specimens using a QIAamp DSP Virus (QIAGEN GmbH, Hilden, Germany) Kit, according to the manufacturer’s instructions, and the quantity and quality of the isolated RNA was determined by a NanoDrop spectrophotometer (Thermo Scientific, Wilmington, MA, USA) instrument.

### SARS-CoV-2 genome amplification using real time PCR

The real-time polymerase chain reaction (RT-PCR) method was used for the detection of the genomic RNA of SARS-CoV-2 in the isolated RNA using the Rotor-Gene Q (QIAGEN, Germany) instrument. In the current study a conserved region of RdRp (RNA-dependent RNA polymerase.) and E (envelope) gene of the SARS-CoV-2 were amplified, as described previously in detail [30]. For negative and positive controls, the samples of 10 healthy people and 10 individuals with SARS-CoV-2 infection were used, respectively.

### Statistical analysis

Statistical analysis was performed by SPSS software version 20 (SPSS Inc., Chicago, IL, USA). For evaluation of the normality of the data the Kolmogorov-Smirnov test was used, and also the statistical differences between categorical variables were examined by Fisher exact test or Chi-square test, as appropriate. It is noteworthy that, a P-value less than 0.05 (P < 0.05) was considered statistically significant.

## Results

From January 2020 to August 2020, a total of 25,044 consecutive Iranian were included in this cross sectional study. Of those studied, 98 (0.40%) were neonates born to mothers or relatives with Covid-19, and the current research was performed on these neonates (Fig. 1). The mean age of the neonates at the time of sampling was 4.7 ± 6.9 days (a range of: 1–28 days). Of the 98 neonates, 49 (50.0%) were male. Demographic, and laboratory data for the studied neonates and their mothers are presented in Table 1.

**Table 1:** Demographic parameters of all participants, neonates and mothers of this survey

Results		Male	Female	Total	P. Value
Parameters					
Age of all participants/days or years at the time of sampling		41.8 ± 14.5 (1 day-91 years)	42.9 ± 14.9 (1 day-93 years)	42.5 ± 14.7 (1 day-93 years)	0.404 Student T Test
PCR results of SARS-CoV-2 for all participants	Positive	3717 (27.7%)	3034 (26.1%)	6751 (27.0%)	0.0036* Chi-Square Test
	Negative	9692 (72.3%)	8601 (73.9%)	18,293 (73.0%)	
	Total	13,409 (53.5%)	11,635 (46.5%)	25,044 (100.0%)	
Age of neonates/days at the time of sampling		5.2 ± 7.8 (1–28 days)	4.1 ± 5.7 (1–27 days)	4.7 ± 6.9 (1–28 days)	0.956 Mann-Whitney U Test
Age groups of neonates (days)	1–10	43 (87.8%)	46 (93.9%)	89 (90.8%)	0.500 Chi-Square Test
	11–28	6 (12.2%)	3 (6.1%)	9 (9.2%)	
PCR results of SARS-CoV-2 of all neonates	Positive	6 (12.5%)	0 (0.0%)	6 (6.1%)	0.013^a^ Chi-Square Test
	Negative	43 (87.5%)	49 (100.0%)	92 (93.9%)	
Age of mothers/years at the time of sampling		–	–	30.8 ± 5.1 (17–41 years)	–
Age groups of mothers (years)	15–20	–	–	4 (4.1%)	–
	21–25	–	–	12 (12.2%)	
	26–30	–	–	32 (32.7%)	
	31–35	–	–	30 (30.6%)	
	35–40	-	–	18 (18.4%)	
	> 40	–	–	2 (2.0%)	

^a^Statistically significant

The RNA of SARS-CoV-2 was detected in the oropharyngeal samples from 6 (6.1%) of the 98 studied neonates. As a result, these neonates had Covid-19 infection. It is noteworthy that in these 6 neonates with Covid-19 disease, the mother of 3 (50.0%) neonates, the mother and father of 2 (33.3%) neonates and the grandmother of one (16.7%) neonate was infected with the SARS-Co-2.

Also, the genomic-RNA of this virus was detected in the nasopharyngeal and oropharyngeal specimens from 25 (25.5%) of the 98 studied mothers (these mothers were included in the initial screening). The demographic and epidemiological characteristics of the studied neonates and mothers of this research are shown in Tables 2 and 3. All the information about Iranian newborns with COVID-19 Infection are summarized in Table 4.

**Table 2:** The Demographic and Epidemiological Characteristics of the Studied Neonates of this Survey

Parameters		Positive	Negative	Total	P value
No		6 (6.1%)	92 (93.9%)	98 (100.0%)	–
Age of neonates/days at the time of sampling		2.8 ± 2.0 (1–5 days)	4.8 ± 7.0 (1–28 days)	4.7 ± 6.8 (1–28 days)	0.274 Mann-Whitney U Test
Age groups of neonates (Days)	1–10	6 (100.0%)	83 (90.2%)	89 (90.8%)	0.633 Chi-Square Test
	11–28	0 (0.0%)	9 (9.8%)	9 (9.8%)	
Neonatal maturity	Mature	5 (83.3%)	76 (82.6%)	81 (82.7%)	0.722 Chi-Square Test
	Immature	1 (16.7%)	16 (17.4%)	17 (17.3%)	
Epidemiological characteristics of neonates					
None		2 (33.3%)	51 (55.4%)	53 (54.1%)	0.409 Fisher’s exact Test
Fever		4 (66.7%)	11 (12.0%)	15 (15.3%)	0.005^a^ Fisher’s exact Test
General weakness		0 (0.0%)	4 (4.3%)	4 (4.1%)	1.000 Fisher’s exact Test
Dyspnea		2 (33.3%)	23 (25.0%)	25 (25.5%)	0.643 Fisher’s exact Test
Confusion		0 (0.0%)	2 (2.2%)	2 (2.0%)	1.000 Fisher’s exact Test
Dry cough		1 (16.7%)	3 (3.3%)	4 (4.1%)	0.226 Fisher’s exact Test
Tachycardia		1 (16.7%)	0 (0.0%)	1 (1.0%)	0.061 Fisher’s exact Test
Cardiovascular disease		0 (0.0%)	1 (1.1%)	1 (1.0%))	1.000 Fisher’s exact Test

^a^Statistically significant survey

**Table 3:** The demographic and epidemiological characteristics of the studied mothers of this survey

Parameters		Positive	Negative	Total	P. value
No		25 (25.5%)	73 (74.5%)	98 (100%)	–
Age of mothers/Years at the time of sampling		30.2 ± 5.7 (19–39)	30.9 ± 5.1 (17–41)	30.8 ± 5.1 (17–41)	0.533 Student T Test
Age groups of mothers (Years)	15–20	1 (4.0%)	3 (4.1%)	4 (4.1%)	0.443 Chi-Square Test
	21–25	6 (24.0%)	6 (8.2%)	12 (12.2%)	
	26–30	6 (24.0%)	26 (35.6%)	32 (32.7%)	
	31–35	6 (24.0%)	24 (32.8%)	30 (30.6%)	
	36–40	6 (24.0%)	12 (16.4%)	18 (18.4%)	
	> 40	0 (0.0%)	2 (2.7%)	2 (2.0%)	
Epidemiological characteristics of mothers					
None		8 (32.0%)	60 (82.2%)	68 (69.4%)	< 0.001^a^Fisher’s exact Test
Fever		12 (48.0%)	9 (12.3%)	21 (21.4%)	< 0.001 ^a^Fisher’s exact Test
Chills		10 (40.0%)	7 (9.6%)	17 (17.3%)	< 0.001 ^a^Fisher’s exact Test
General weakness		6 (24.0%)	3 (4.1%)	9 (9.2%)	< 0.001 ^a^Fisher’s exact Test
Skeletal pain		3 (12.0%)	1 (1.4%)	4 (4.1%)	0.016 ^a^Fisher’s exact Test
Hypertension		1 (4.0%)	1 (1.4%)	2 (2.0%)	0.410 Fisher’s exact Test
Dyspnea		1 (4.0%)	3 (4.1%)	4 (4.1%)	0.228 Fisher’s exact Test
Dry cough		5 (20.0%)	5 (6.8%)	10 (10.2%)	0.007 ^a^Fisher’s exact Test
Tachycardia		0 (0.0%)	1 (1.4%)	1 (1.0%)	0.568 Fisher’s exact Test
Acute respiratory disease		1 (4.0%)	1 (1.4%)	2 (2.0%)	0.013 ^a^Fisher’s exact Test
Decreased sense of smell		6 (24.0%)	2 (2.7%)	8 (8.2%)	< 0.001 ^a^Fisher’s exact Test
Decreased sense of taste		6 (24.0%)	2 (2.7%)	8 (8.2%)	< 0.001 ^a^Fisher’s exact Test

^a^Statistically significant

**Table 4:** Complete information about Iranian Newborns with COVID-19 Infection

Neonates							Mothers		
No.	Age/days^a^	Gender	Maturity	Hospitalization	Prognosis	Clinical Characteristics	Aage/\years^a^	PCR result	Clinical manifestations
8	5	Male	Mature	Yes/NICU	Survived	Fever, Tachycardia	29	Positive	Fever, General Weakness, Skeletal pain
13	1	Male	Immature	Yes/NICU	Died	Dyspnea	29	Positive	Dyspnea, Skeletal pain, Dry cough
58	1	Male	Mature	Yes/NICU	Survived	Fever	30	Negative	Hypertension, General Weakness, Dry cough
61	5	Male	Mature	Yes/NICU	Survived	Fever, Dry cough	36	Positive	Fever, Chills, Dry cough
75	2	Male	Mature	Yes/NICU	Survived	Fever	31	Positive	Fever, Chills, Acute respiratory disease, Dry cough, Decreased sense of smell and taste
94	1	Male	Mature	Yes/NICU	Survived	Fever, Dry cough	39	Negative	Decreased sense of smell and taste

^a^At the time of sampling

Positive PCR results of SARS-CoV-2 for all participants was observed 6751 (27.0%). Out of 25,044 (100.0%) all participants, 13,409 (53.5%) were male. The mean age of the neonates’ mothers was 30.6 ± 5.3 years (a range of: 17–41 years). Out of 49 (50.0%) male, positive and negative PCR results of SARS-CoV-2 for neonates was detected 6 (12.5%) and 43 (87.5%), respectively. Out of 49 (50.0%) female, positive and negative PCR results of SARS-CoV-2 for neonates was detected 0 (0.0%) and 49 (100.0%), respectively (Table 1). Out of the 98 neonates, 81 (82.7%) were mature and 17 (17.3%) were immature. Out of 81 (82.7%) mature neonates, positive and negative PCR results of SARS-CoV-2 was detected 5 (6.2%) and 76 (82.6%), respectively. Out of 17 (17.3%) immature neonates, positive and negative PCR results of SARS-CoV-2 was detected 1 (16.7%) and 16 (17.4%), respectively (Table 2).

In this research, a significant association was observed between PCR results of SARS-CoV-2 for all participants and gender (*P* = 0.0036, Chi-Square Test). No statistically significant association was found between age of neonates/days as well as age groups of neonates (days) and gender. In addition, a significant association was observed between PCR results of SARS-CoV-2 of all neonates and gender (Table 1). No statistically significant association was observed between PCR results of SARS-CoV-2 of all neonates and age of neonates/days as well as age groups of neonates (days). Also, no statistically significant association was observed between neonatal maturity and PCR results of SARS-CoV-2 of all neonates. Furthermore, a significant association was observed between PCR results of SARS-CoV-2 of all neonates and fever, tachycardia as well as asymptomatic neonates (none). While, No statistically significant association was found between PCR results of SARS-CoV-2 of all neonates and general weakness, dyspnea, confusion, dry cough and cardiovascular disease (Table 2).

Also, no statistically significant association was observed between PCR results of SARS-CoV-2 for neonates’ mothers and age of mothers/years as well as age groups of mothers (years). In addition, a significant association was observed between PCR results of SARS-CoV-2 for neonates’ mothers and fever, chills, general weakness, skeletal pain, dry cough, acute respiratory disease, decreased sense of smell, decreased sense of taste as well as asymptomatic mothers (none). While, no significant association was observed between PCR results of SARS-CoV-2 for neonates’ mothers and hypertension, dyspnea and tachycardia (Table 3).

## Discussion

Several factors, such as physical contact, respiratory droplets, and spread of aerosols, lead to individual-to-individual transmission of COVID-19 [31, 32]. In this study, out of 98 neonates born to mothers or all those who live together in the same house and are in contact with the neonates with Covid-19, 6 (6.1%) cases were confirmed with SARS-CoV-2 infection, which had a positive result for polymerase chain reaction (PCR). It is not clear whether the transmission of SARS-CoV-2 infection occurred due to the vertical transmission from mother to neonate.

In December 2019, the spread of SARS-CoV-2 infection had rapidly increased worldwide. Initially, most cases were reported in older patients. After a while, neonates were reported to be infected with SARS-CoV-2. According to the latest data, individuals with the age of < 19 years accounted for 1–5% of the SARS-CoV-2 infection. Although mortality is rare in this age group, younger children are likely to develop severe illness [6, 33–35]. Children infected with the virus show milder symptoms than adults [34]. Although the COVID-19 infection has been reported in neonates, mother-to-infant perinatal and vertical transmission of SARS-CoV-2 has not been confirmed to date [3, 34, 36–40]. One of the most important consequences of viral infections occurring during pregnancy is the intrauterine transmission. TORCH pathogens, such as toxoplasmosis, rubella, herpes simplex virus (HSV), cytomegalovirus (CMV), other Infections (parvovirus B19, varicella-zoster virus [VZV], and syphilis), human immunodeficiency virus (HIV), and Hepatitis viruses, ebola virus, and zika virus, can be transmitted from mother to fetus and infect the fetus [41]. Generally, the vertical transmission of viral infection occurs through the placenta and blood. However, this route of transmission has been demonstrated not to occur among pregnant women by SARS-CoV and MERS-CoV. Nevertheless, infection with these coronaviruses can cause severe pneumonia in mothers [42]. The SARS-CoV-2 can affect newborns in several ways, including transmission through horizontal or vertical ways, through causing infection, by maternal SARS-CoV-2 infections (e.g., preterm neonates) [43]. One study demonstrated that early infection among newborns was commonly mild and uncommon [43]. According to the WHO recommendations, this manner is not recommended for the separation of mother and newborn infant because several harmful consequences arise for the relationship between mother and neonate [28, 44, 45].

This cross-sectional study was performed on the clinical and epidemiological characteristics and neonatal and maternal results in the case of neonates born to mothers or relatives with COVID-19. To date, a limited number of studies have reported COVID-19 among pregnant women infected with SARS-CoV-2 [31]. The ribonucleic acid (RNA) of SARS-CoV-2 was detected in the oropharyngeal specimens of 6 (6.1%) subjects out of the 98 studied neonates. Moreover, the genomic RNA of SARS-CoV-2 was detected in the nasopharyngeal, and oropharyngeal samples of 25 (25.5%) cases out of the 98 studied neonates’ mothers.

This study, according to clinical manifestations, demonstrated the prevalent symptoms of these mothers with positive SARS-CoV-2 infection during COVID-19, including fever (n = 12; 48.0%), chills (n = 10; 40.0%), asymptomatic (n = 8; 32.0%), general weakness (n = 6; 24.0%), decreased sense of smell (n = 6; 24.0%), decreased sense of taste (n = 6; 24.0%), and dry cough (n = 5; 20.0%), respectively. In addition, among these neonates’ mothers, there were less common symptoms, such as skeletal pain (n = 3; 12.0%), hypertension (n = 1; 4.0%), dyspnea (n = 1; 4.0%), acute respiratory disease (n = 1; 4.0%), and tachycardia (n = 0; 0.0%), respectively. Furthermore, according to demographic and epidemiological characteristics, the common symptoms of these neonates with positive SARS-CoV-2 infection included fever (n = 4; 66.6%), and dyspnea (n = 2; 33.3%), respectively. Furthermore, among these neonates, there were less common symptoms, such as asymptomatic (n = 2; 33.3%), dry cough (n = 1; 16.7%), tachycardia (n = 1; 16.7%), cardiovascular disease (n = 0; 0.0%), general weakness (n = 0; 0.0%), and confusion (n = 0; 0.0%), respectively. The present findings seem to be consistent with the findings of other studies conducted in Wuhan, China, demonstrating fever as the common symptom among these mothers with positive SARS-CoV-2 infection [31, 46].

Severe acute respiratory syndrome (SARS) and SARS-CoV-2 have been reported to be 79% similar in sequence [47]. ). In the SARS infection, the rate of mortality is reported to be 10% [48]. In addition, among pregnant women, the mortality rate of SARS infection was reported as 25% [20]. In some previous studies, no cases of perinatal infection and disease have been reported among neonates born to mothers with the SARS infection [20, 49]. In some studies, the mortality rate among patients with COVID-19 has been reported to be approximately 1.4% [50].

To date, different case reports have demonstrated positive SARS-CoV-2 results within 48 h among newborns. One study reported positive SARS-CoV-2 neonates (3/33) with positive SARS-CoV-2 mothers [51]. In the UK, six neonates were positive for SARS-CoV-2 infection [52]. Another study reported a SARS-CoV-2-positive preterm newborn with a mother with a severe form of COVID-19 and positive SARS-CoV-2 for amniotic fluid [53]. Positive SARS-CoV-2 for placental tissue was evaluated in a mother with symptoms of cough and fever during delivery. In addition, there was a report of a positive SARS-CoV-2 result for the neonate [9]. In Wuhan, China, out of seven pregnant women, three neonates were positive for SARS-CoV-2 infections, and the SARS-CoV-2 infection was observed in one neonate about 36 h after birth. The clinical characteristics among these pregnant women were reported as fever (n = 6; 86%), cough (n = 1; 14%), shortness of breath (n = 1; 14%), and diarrhea (n = 1; 14%), respectively [31]. According to a previous study performed in Wuhan, China, COVID-19 occurred in one neonate 36 h after birth; on the other hand, SARS-CoV-2 tests for cord blood and placenta in this neonate were reported as negative. As a result, it seems that the SARS-CoV-2 infection is probably not caused by vertical intrauterine transmission [31].

In one study, the rate of neonates with SARS-CoV-2 infection was reported as 7.4%. Their mothers were also positive for the SARS-CoV-2 infection [54]. In China, a neonatal case with the SARS-CoV-2 infection was reported [36]. In another study, all neonatal samples for the detection of SARS-CoV-2 were negative [39]. In China, 9 mothers with the SARS-CoV-2 infection were diagnosed by the evaluation of clinical features, and 10 mothers with the SARS-CoV-2 infection were confirmed by laboratory tests. In stool, urine, gastric fluid, and throat swab samples, PCR results for SARS-CoV-2 were negative among all neonates. However, in the throat swab specimen, PCR results for SARS-CoV-2 were positive for one neonate. The test of the throat swab specimen was repeated with a false-positive result. Consequently, SARS-CoV-2 was not detected in the umbilical cord blood and amniotic fluid [46].

This study has some limitations. It seems that screening participants for a short period could not clearly show vertical transmission from mother to newborn or transmission through other routes. The study results of one population at the time of the pandemic may differ from the results of other studied populations.

## Conclusion

The findings of this study demonstrated that the rate of COVID-19 among neonates born to mothers or all those who live together in the same house and are in contact with the neonates with SARS-CoV-2 infection in the Iranian population is about 6.1%. The clinical and epidemiological characteristics of these pregnant women with the SARS-CoV-2 infection during pregnancy were approximately similar to those of pregnant women without SARS-CoV-2 infection. The transmission of SARS-CoV-2 infection due to vertical transmission from mother to neonate is not well understood. Therefore, it is required to perform further studies to evaluate the epidemiological and clinical characteristics of maternal and neonatal outcomes of pregnant mothers with SARS-CoV-2 infection during pregnancy and transmission of COVID-19 through vertical transmission from pregnant mother to neonate. It should be noted that vertical infection of SARS-CoV-2 can be proven by investigating multiple specimens such as nasopharynx, cord blood, and amniotic fluid in the early time of birth.

## Data Availability

The datasets analyzed and/or used during the present survey are available on request from the responsible author.
